# Healing of Osteochondral Defects Implanted with Biomimetic Scaffolds of Poly(ε-Caprolactone)/Hydroxyapatite and Glycidyl-Methacrylate-Modified Hyaluronic Acid in a Minipig

**DOI:** 10.3390/ijms19041125

**Published:** 2018-04-09

**Authors:** Yi-Ho Hsieh, Bo-Yuan Shen, Yao-Horng Wang, Bojain Lin, Hung-Maan Lee, Ming-Fa Hsieh

**Affiliations:** 1 Department of Biomedical Engineering, Chung Yuan Christian University, 200 Chung Pei Road, Chung-Li District, Taoyuan City 320, Taiwan; dilantin11@gmail.com (Y.-H.H.); linbojain@gmail.com (B.L.); hml@hinet.net (H.-M.L.); 2 Department of Orthopedics, Min-Sheng General Hospital, 168, Ching Kuo Road, Taoyuan 330, Taiwan; 3 Mater Program for Nanotechnology, Chung Yuan Christian University, 200 Chung Pei Road, Chung-Li District, Taoyuan City 320, Taiwan; dunshore1@gmail.com; 4 Department of Nursing, Yuanpei University of Medical Technology, 306, Yuanpei Street, Hsinchu 300, Taiwan; pigmodel@gmail.com; 5 Department of Orthopedics, Taoyuan Armed Forces General Hospital, No. 168, Zhongxing Road, Longtan District, Taoyuan City 325, Taiwan; 6 Department of Orthopedics, Hualien Tzu Chi General Hospital, No. 707, Sec. 3, Chung Yang Road, Hualien 970, Taiwan

**Keywords:** cartilage, biomimetic, fused deposition modeling, poly(ε-caprolactone), hyaluronic acid, large animal models

## Abstract

Articular cartilage is a structure lack of vascular distribution. Once the cartilage is injured or diseased, it is unable to regenerate by itself. Surgical treatments do not effectively heal defects in articular cartilage. Tissue engineering is the most potential solution to this problem. In this study, methoxy poly(ethylene glycol)-block-poly(ε-caprolactone) (mPEG-PCL) and hydroxyapatite at a weight ratio of 2:1 were mixed via fused deposition modeling (FDM) layer by layer to form a solid scaffold. The scaffolds were further infiltrated with glycidyl methacrylate hyaluronic acid loading with 10 ng/mL of Transforming Growth Factor-β1 and photo cross-linked on top of the scaffolds. An in vivo test was performed on the knees of Lanyu miniature pigs for a period of 12 months. The healing process of the osteochondral defects was followed by computer tomography (CT). The defect was fully covered with regenerated tissues in the control pig, while different tissues were grown in the defect of knee of the experimental pig. In the gross anatomy of the cross section, the scaffold remained in the subchondral location, while surface cartilage was regenerated. The cross section of the knees of both the control and experimental pigs were subjected to hematoxylin and eosin staining. The cartilage of the knee in the experimental pig was partially matured, e.g., few chondrocyte cells were enclosed in the lacunae. In the knee of the control pig, the defect was fully grown with fibrocartilage. In another in vivo experiment in a rabbit and a pig, the composite of the TGF-β1-loaded hydrogel and scaffolds was found to regenerate hyaline cartilage. However, scaffolds that remain in the subchondral lesion potentially delay the healing process. Therefore, the structural design of the scaffold should be reconsidered to match the regeneration process of both cartilage and subchondral bone.

## 1. Introduction

Articular cartilage is structurally deficient in vascular distribution and cannot regenerate once the cartilage is injured [1]. The uneven articular surface will gradually develop into arthritis over time, and this process is irreversible. Thus, treating blemishes of joint surfaces has been a major challenge for orthopedic surgeons. Current surgical approaches have been developed and range from arthroscopic ablation, osteotomy, and arthroplasty to cartilage restoration such as microfracture surgery, osteochondral autografts (mosaicplasty), autologous chondrocyte implantation (ACI), and fresh osteochondral allograft. However, these treatments do not fully heal the bone and cartilage tissue, and the regenerated cartilage is of inferior quality [2].

Microfracture surgery is a marrow stimulation technique that involves the migration of mesenchymal stem cells (MSCs) from the bone marrow to the defective site. This technique results in fibrocartilage regeneration and the mechanical property is inferior to native hyaline cartilage [3]. Mosaicplasty is a surgical procedure involving transplantation of cylindrical osteochondral grafts from a non-weight bearing region of the knee to the defects. The limitation of this technique is considered the scarce donor tissue source and donor site morbidity [4]. ACI is a cell-based approach for treating cartilage lesions, where autologous chondrocytes are implanted into the cartilage defect region. However, it has been reported that full restoration of the functional knee cannot be achieved [5]. In cartilage injury or osteoarthritis of joints, subchondral bone changes are a distinctive feature and can consist in, for example, sclerosis, cyst formation, bone attrition, bone marrow lesions, osteophytes, and subchondral bone remodeling [6]. Therefore, subchondral bone should be taken into consideration in the treatment of cartilage defects.

The application of tissue engineering to the repair of defects in cartilage and subchondral bone may serve as a solution to the above-mentioned inadequacies in treatment [7]. Tissue engineering to promote cartilage regeneration must take on three elements: scaffolds, cells, and growth agents [8,9,10]. Scaffolds can provide cell adhesion and proliferation. The scaffold material selection, mechanical properties, and pore architecture of the scaffold can affect the cell growth following implantation. Rapid prototyping (RP) can be used for scaffold fabrication, with various geometrical structures, controllable pore size and porosity, connectivity, and other advantages [11,12]. With RP, scaffolds that closely match defect shapes can be constructed. Poly(ε-caprolactone) (PCL) has good biocompatibility and has been employed in the fused deposition modeling (a type of RP) of porous scaffolds [13,14]. However, because of the synthetic nature of PCL, the cells are not prone to adhere to and grow on the surface of PCL scaffolds. It has been found that Arginylglycylaspartic acid (RGD) peptide can enhance cell adhesion by binding the integrin αvβ3 in cell membranes. The use of RGD peptide grafted to the surface of PCL material promotes cells adhesion [15,16]. To enhance osteointegration and mimic the bony environment, hydroxyapatite (HAp) is typically added to the scaffold [17]. To mimic the cartilaginous environment, hyaluronic acid (HA) combined with transforming growth factor β1 (TGF-β1) can induce the differentiation of bone marrow mesenchymal stem cells to hyaline cartilage [18,19]. In consideration of both scaffold design and anatomical structure, glycidyl methacrylate was grafted to hyaluronic acid (GMHA), making a photo-curable hydrogel to carry growth factors [20]. In the present study, a solution containing TGF-β1 was photo-crosslinked to form a hydrogel layer fixed on the upper side of an RP scaffold. With the biphasic-composite-designed scaffold, both the injured articular cartilage and subchondral bone were repaired and regrown.

Before bringing out such treatments into clinical practice, in vivo animal studies are required to close the gap between in vitro experiments and human clinical studies. In animal studies, the size of the joint and cartilage thickness are essential for simulating human disease. The animal size roughly corresponds to the size of the joint and cartilage thickness. In general, human cartilage lesions requiring treatment are at least 10 mm in diameter. The knee joints of New Zealand White rabbits are large enough to create defects of only about 3–4 mm, and the cartilage is relatively thin. Minipigs have larger knee joints such that 10 mm defects can be induced, and they have limited capability for the endogenous repair of chondral and osteochondral defects, which is similar to humans [21,22,23].

## 2. Results

The minipigs were sacrificed one year after scaffold implantation. Histological sections of the experimental group showed good regeneration of cartilage and subchondral bone in the defect area. The sections of the newly formed cartilage stained with hematoxylin and eosin presented a loose arrangement chondrocytes located in the lacunae surrounded by an extracellular matrix, which is a feature of hyaline cartilage. Beneath the cartilage layer, a good regeneration of the subchondral bone was noted. Undegraded scaffold in the deep part of the femur condyle as well as bone tissue growth in the pores of the scaffold was observed. In the control group, the defect region was observed to be filled with hypertrophic cartilage with invasion to the subchondral bone area, and the tissue’s histology and morphology showed that the newly formed cartilage was fibrocartilage instead of hyaline cartilage.

### 2.1. Live Magnetic Resoance Imaging (MRI) Monitoring of Articular Cartilage Repair in Minipigs

**Figure 1:**
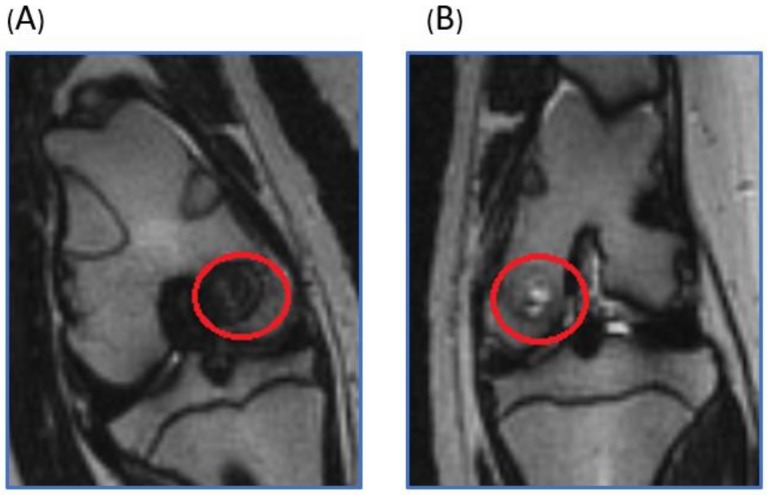
T2 weighted coronal plane MRI. The red circle was the operation area. (**A**) In the scaffold implantation group, the bone defect was fully filled by the scaffolds and tissue ingrowth. (**B**) In the control group, the bone defect region was filled with joint fluid, and partial bony ingrowth was noted.

Under general anesthesia, the magnetic resonance imaging (MRI) of the minipigs was performed six months after scaffold implantation. In the experimental knee joint, the bone defect was fully filled without free fluid. The scaffold could still be identified and maintained its structure. In the control, the bone defect region was filled with joint fluid, and partial bony ingrowth was noted (Figure 1).

### 2.2. Macroscopic Observation

**Figure 2:**
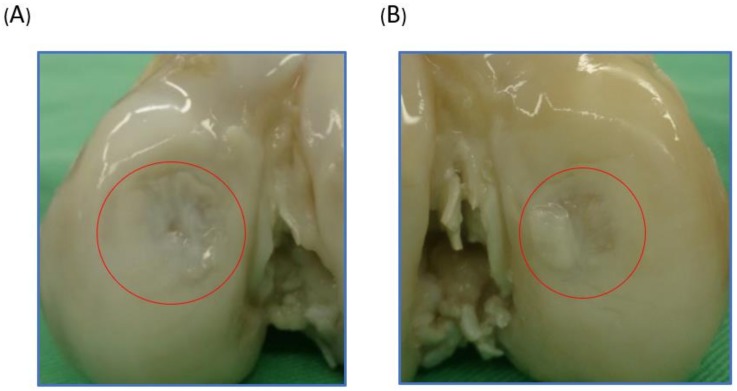
Gross observation of the repair of swine knees implanted with biphasic scaffolds of Poly(ε-caprolactone)/Hydroxyapatite and glycidyl-methacrylate-modified hyaluronic acid or without implants as the control group one year after surgery. The red circle was the operation area. (**A**) The scaffold implantation group showed successful regeneration of the previously removed cartilage. (**B**) The control group without implants showed soft tissue hypertrophy and cartilage defect.

Macroscopic observation provided a direct assessment of the cartilage defect repair. One year after engraftment, the minipigs were sacrificed, and their knees were harvested and analyzed. In the experimental group, near full-thickness defects were repaired with glossy white tissue (Figure 2A). The defects in the control group remained largely unfilled (Figure 2B). The International Cartilage Repair Society (ICRS) macroscopic score for the scaffold implantation group in terms of defect filling (3), integration to surrounding host cartilage (3), and macroscopic appearance (3) were all higher than those of the control group (3,1,1). The overall repair assessment of the experimental group was near normal (9, grade II), and the control group was abnormal (5, grade III). 

**Figure 3:**
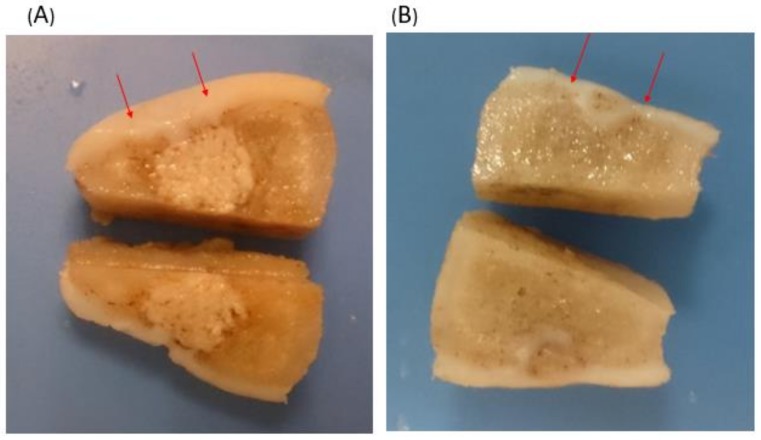
Cross-sectional view of the knee joint of the experimental group with scaffold implantation and the control group without implants. The operation area was between the red arrows. (**A**) With scaffold implantation, the defect of the surface was fully filled with hyaline cartilage, and subchondral bone regeneration was noted. Undegraded scaffold was noted in the deep layer. (**B**) Without implants, the defect region was filled with hypertrophic cartilage-like soft tissue with invasion to the subchondral bone area.

To observe the repair tissue at the defect region beneath the surface of the regenerated cartilage, each condyle was sectioned along the sagittal and frontal plane. In the experimental group, the cartilage layer and subchondral bone were successfully regenerated, and undegraded scaffold in the deep part of the femur condyle was noted (Figure 3A). In the control group, the defect site was observed to be filled with hypertrophic cartilage-like tissue with invasion to the subchondral area (Figure 3B).

### 2.3. Histological Analysis

**Figure 4:**
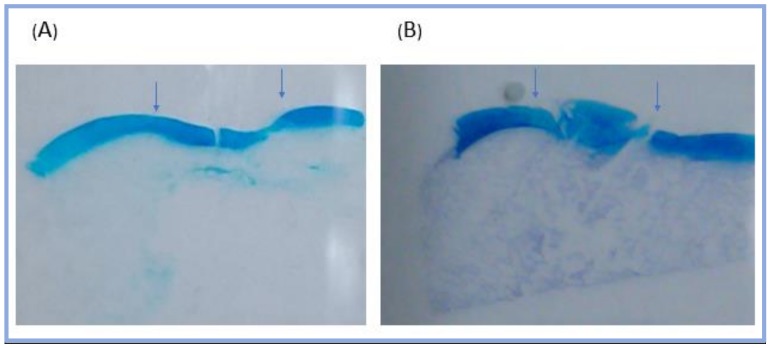
Alcian blue staining of the minipig knee joints one year after operation. The operation area was between the arrows. (**A**) The experimental group showed a smooth surface and an even thickness of the regenerated cartilage. (**B**) The control group presented a discontinuous and hypertrophic cartilage surface, and the subchondral bone area was replaced by cartilage-like soft tissue.

To identify the quality of the regenerated tissue, the experimental group and the control group were histologically evaluated. One year after implantation, the cartilage of the scaffold implantation group was successfully regeneration. Alcian blue staining was used to detect the presence of glycosaminoglycans, which parallels the degree of cartilage formation. The Alcian blue staining of the experimental group displayed an even concentrated blue color, the thickness was close to that of the surrounding normal cartilage, and the surface was smooth and uniform. The control group presented discontinuous and hypertrophic cartilage surface, with invasion to the subchondral bone area (Figure 4).

**Figure 5:**
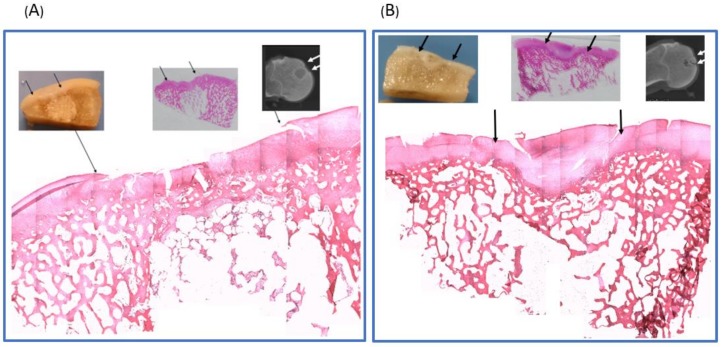
(**A**) Hematoxylin & Eosin staining of the biphasic scaffold implantation group (reorganized picture, original magnification ×5). The histology showed successfully cartilage regeneration over defect creating region (between two arrows). The repaired cartilage integrated well with the surrounding cartilage. Good growth of the subchondral bone was observed. The defect in the lower panel was filled with undegraded scaffold, and partial bony ingrowth in the scaffold porous was noted. (**B**) H & E staining of the untreated control group. Untreated defects in the control group were filled mostly with disorganized fibrocartilage that did not restore a smooth articular surface with adjacent host cartilage. The subchondral bone region was replaced by fibrocartilage.

The second staining method, hematoxylin and eosin (H & E) staining, was used to show the distribution of the nucleus and cytoplasmic inclusions. The experimental group was shown in Figure 5A. Along the upper panel, cartilage successfully regenerated, and the displayed tissue morphology was resembled normal hyaline cartilage. The regenerated cartilage, compared to that of the control group, showed better columnar organization and integrated well with the surrounding cartilage. Good growth of the subchondral bone was also observed. However, the lower panel of the defect was filled with undegraded scaffold, with a partial ingrowth of bone tissue into the scaffold porous. In contrast, untreated defects in the control group (Figure 5B) were filled mostly with disorganized fibrocartilage that did not restore a continuous articular surface with adjacent host cartilage. The subchondral bone region was filled with fibrocartilage. These results suggest that an mPEG-PCL porous scaffold combined with GMHA hydrogel loading with TGF-β1 can help cartilage and subchondral bone formation and prevent fibrous tissue invagination.

**Figure 6:**
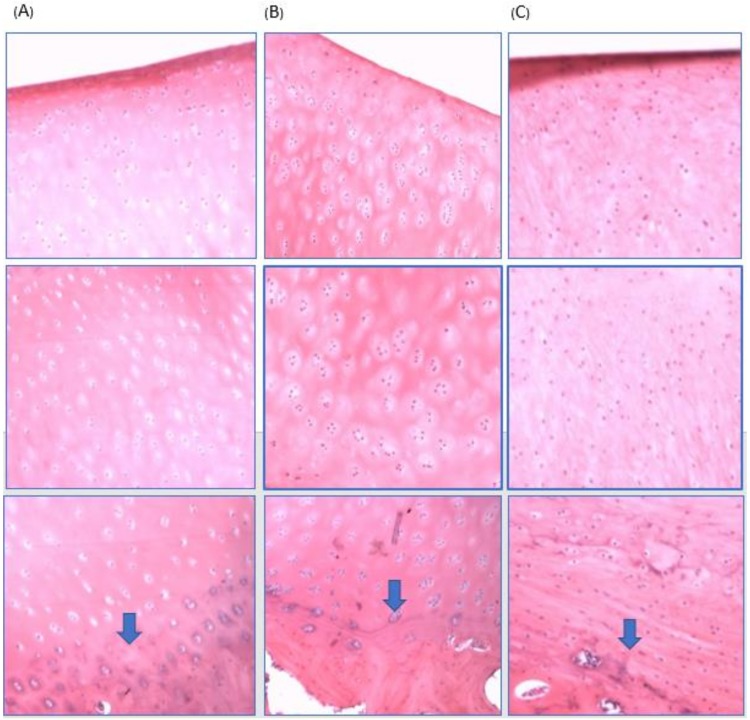
H & E staining (original magnification ×100) of (**A**) The normal minipig knee cartilage showed native hyaline cartilage cell morphology with round chondrocytes in the lacunae surrounding by the matrix. The tidemark is indicated by blue arrows. (**B**) The cell morphology of the regenerated cartilage from the biphasic scaffold implantation group was similar to the native hyaline cartilage with round chondrocytes in lacunae. The tidemark presented well. (**C**) The control group showed only spindle-shaped, fibroblast-like cells within the regenerated cartilage. The tidemark between calcified and uncalcified cartilage could not be well distinguished.

To identify the regenerated tissue type, the cell morphology and matrix structure were analyzed. H & E staining of the normal minipig knee cartilage, the experimental group cartilage, and the control group cartilage were analyzed. The normal minipig knee cartilage showed native hyaline cartilage cell morphology with round chondrocytes in the lacunae surrounded by the matrix. A tidemark between cartilage and subchondral bone was observed (Figure 6A). Chondrocytes in the tissue regenerated from the biphasic scaffold implantation group were round, clustered, and surrounded by a matrix that is similar to those found in the surrounding hyaline cartilage. However, there were more chondrocytes in the lacunae than that of native hyaline cartilage, suggesting that the regenerated cartilage was hyaline cartilage albeit not completely differentiated (Figure 6B). In the defect-only control group, an abundance of spindle-shaped, fibroblast-like cells within the defect site was observed, suggesting that only fibrous or fibrocartilaginous tissues were formed (Figure 6C). Moreover, the tidemark between calcified and uncalcified cartilage was clear in the experimental group, but that in the control group could not be well distinguished.

**Figure 7:**
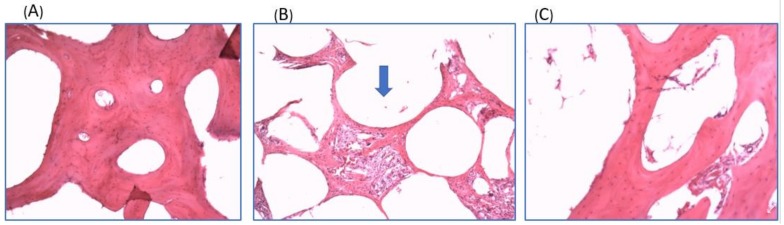
H & E staining (original magnification ×40) of (**A**) The normal minipig knee cancellous bone tissue showing the characteristic structure of cancellous bone with trabecular structure and numerous spaces containing bone marrow. (**B**) The experimental group showed undegraded scaffold (blue arrow) one year after implantation. The scaffold porous was filled with mixed bone tissue and fibrotic tissue. (**C**) The regenerated bone tissue of control group showed loose trabecular structure.

The regenerated bone tissue was also analyzed. With H & E staining, the normal minipig knee cancellous bone tissue showing the characteristic structure of cancellous bone with trabecular structure and numerous spaces containing bone marrow (Figure 7A). In the biphasic scaffold implantation group, the lower panel of the defect was filled with undegraded scaffolds. The scaffold porous was filled with mixed bone tissue and fibrotic tissue (Figure 7B). In the control group, the regenerated bone tissue showed a loose trabecular structure (Figure 7C).

An ICRS Visual Histological Assessment was used to quantitatively compare the regenerative cartilage from the experimental group with that from the control group. For surface smoothness, the experimental group scored 2.5 and the control group scored 0.5, suggesting that the experimental group had a smoother and integrated cartilage surface. For matrix types, the experimental group scored 2 and the control group scored 0.5, indicating greater hyaline cartilage formation in the experimental group. In terms of cell distribution, the experimental group presented in a columnar/clustery arrangement with a score of 2, and the control group was more disorganized and presented with the score of 0.5. For cell viability, all two groups scored 2. For subchondral bone evaluation, the repair in the experimental group scored 2.5 and the no-implant group scored 1 due to fibrotic tissue invagination. Finally, for cartilage mineralization, no pathological mineralization was observed in either group, both had a score of 3 (Table 1). Based on the ICRS Visual Histological Assessment, we found that the experimental group can obtain better cartilage and subchondral bone regeneration compared with the control group.

### 2.4. Computed Tomography (CT) Scan Evaluation

**Figure 8:**
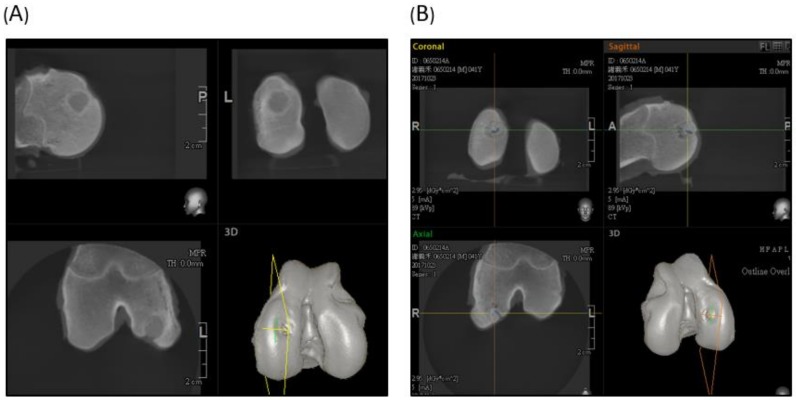
Sagittal, horizontal, and coronal view of the CT scan. (**A**) Experimental group showed an undegraded scaffold in the femur condyle. The articular part of the scaffold degraded well and was replaced by subchondral bone tissue. (**B**) The control group showed partial bony growth in the created defect region.

The CT scan of the experimental group illustrated that the articular side of the scaffold was successfully degraded, subchondral bone regenerated, and the deep area of the created defect was filled with undegraded materials (Figure 8A). In the control group, there was more new bone formation in the defect site, but still some defect without bony growth was noted (Figure 8B).

## 3. Discussion

Impact and torsional joint loading can injure articular cartilage. If joint injuries are localized to the upper layer of the avascular articular cartilage, no inflammation or effective healing can occur. When the lesions are too deep, and the subchondral bone vascular region is injured, the granulation tissue formed to fill the defect then changes to fibrocartilage [24]. Current treatments cannot replace damaged cartilage with new tissue with the same biomechanical properties as normal hyaline cartilage. Subchondral bone changes and remodeling have been observed in cartilage injury or in the osteoarthritis of joints and should be considered in treatment strategies [25]. Several chondrocyte implantation techniques have been used to treat cartilage injury, but mechanical stability cannot be provided by these grafts, and various side effects lead to treatment failure [26,27]. Subchondral bone remodeling and bone replaced by soft tissue and cartilage is another unresolved issue in chondrocyte implantation [28]. The tidemark separating cartilage and subchondral bone cannot be identified well in newly regenerated tissue. Wan-Ju Li et al. [29] noted that biodegradable poly(ε-caprolactone) (PCL) nanofibrous scaffolds seeded with allogeneic chondrocytes or xenogeneic human mesenchymal stem cells (MSCs), implanted to treat chondral defects, repaired the chondral defects, but the subchondral bone was replaced by soft tissue and cartilage. Pulliainen et al. [28] used chondrocyte-seeded poly-l,d-lactic acid scaffolds and found similar results. The phenomenon was similar to that observed in our control group. Chiang et al. [30] reported that fibrous tissue replaced the subchondral bone in a control, no-treatment, defect-created knees, suggesting that fibrous tissue formation is a natural wound-healing process. The recent treatment trend of osteochondral injury is to replace the injured osteochondral lesion by biomaterials, leading to in situ regeneration of not only cartilage but also subchondral bone. Various innovative scaffolds have been designed and reported [31]. Biphasic scaffolds with mimic structures of osteochondral tissues present an opportunity to close this chasm [32]. Frenkel et al. [33] developed biphasic scaffolds using poly(d,l-lactide) (PDLLA) mixed with HAp as an osteogenic layer and polyelectrolytic complex (PEC) hydrogel of HA and chitosan or a collagen type I scaffold as a cartilage layer. However, no growth factors consisted in the devices or the scaffold architecture, and pore size was difficult to control. To solve the problem of scaffold architecture and pore size design, a 3D printing technique was introduced. Sherwood et al. [34] designed 3D-printed scaffolds with an upper cartilage layer consisting of poly(d,l-lactide-*co*-glycolide) and poly(l-lactide) with a porosity of 90% and a lower bone layer composed of poly(l-lactide-*co*-glycolide)/tricalcium phosphate (TCP) with a porosity of 55%. Zhang et al. [35] also used a 3D printing technique to fabricate a biphasic poly(ethylene glycol) (PEG)/β-TCP scaffold. The cartilage layer of a PEG hydrogel was cured on a bone layer of β-TCP as a biphasic scaffold. With 3D printing techniques, architecture and pore size have been designed and controlled, but these studies have not tested for growth factors.

In our study, we demonstrated the successful regeneration of not only articular cartilage but also subchondral bone in an osteochondral defect of a minipig knee joint using biphasic mPEG-PCL porous scaffolds combined with GMHA hydrogel loading with TGF-β1. A tidemark was present, and the subchondral bone was not replaced by soft tissue macroscopically and histologically.

The velocity of degradation depends on the polymers and their molecular weights, as well as other properties. There are four major degradation mechanisms for the biodegradable polymers: hydrolysis, oxidation, enzymatic degradation, and physical degradation [36]. Degradation studies of three-dimensional PCL and PCL-based composite scaffolds have been conducted in vitro (in phosphate buffered saline), and in vivo (rabbit model) studies have revealed only about 7% scaffolds degraded in vivo six months after implantation [37]. In our study, the joint surface side of the mPEG-PCL porous scaffold was degraded and replaced by regenerated cartilage and subchondral bone successfully. The degradation mechanism may be due to hydrolysis by the joint fluid. Histology analysis showed that the scaffold in the deep bone layer was surrounded by regenerated fibrotic tissue and bone tissue. The regenerated tissue may shield the hydrolysis and lead to delayed degradation. The undegraded scaffolds that remain in bone potentially delay the healing process. Therefore, the structural design of the scaffold should be reconsidered to match the regeneration process of both the cartilage and subchondral bone [38].

## 4. Materials and Methods

### 4.1. Polymer Synthesis and Scaffold Fabrication

Raw materials used in the present study include biodegradable methoxy poly(ethylene glycol)-poly(ε-caprolactone) (mPEG-PCL) and hydroxyapatite. The synthesis of mPEG-PCL has been reported in our previous studies [13,39]. The physical and chemical properties and biological toxicity have been analyzed and described in our previous studies and in Supplementary Materials. The theoretical molecular weight of mPEG-PCL in those papers was 9450 Da. The diblock copolymer of mPEG-PCL was synthesized via ring opening polymerization of ε-caprolactone (CL) in the presence of mPEG as a macro-initiator and Sn(Oct)_2_ as a catalyst.

Hyaluronic acid (HA) was employed to carry growth factor (TGF-β1) for the induction of hyaline cartilage in this study. To adhere TGF-β1-loaded HA to the top of the mPEG-PCL scaffold, photo-curable methacrylate groups were attached to HA by a method described in our previous studies. Briefly, 1 g of hyaluronic acid (molecular weight = 4.4 × 10^5^) was dissolved in 100 mL phosphate buffered solution (PBS), and this mixture was stirred overnight. One hundred milliliters of *N*,*N*-dimethylformamide (DMF), 18.04 mL of trimethylamine (TEA), and 35.11 mL of glycidylmethacrylate (GM) were added to the HA solution and stirred for 10 days at room temperature. The resultant solution was then precipitated in acetone at a 10-fold volume with respect to the HA solution, then centrifuged to remove the acetone at 5000 rpm for 10 min. The precipitate was then dissolved in deionized water and subjected to dialysis (molecular weight cut off = 8 kDa) for 24 h and lyophilized to obtain photo-curable GMHA.

The scaffolds were fabricated with fused deposition modeling (FDM) by a custom-made air-pressure-aided deposition system. To improve the cellular attachment properties, mPEG-PCL was reacted with succinic anhydride, whereas the terminal group of the carboxylic acid group was attached to the end group of PCL. It was further grafted with the RGD peptide by condensation.

The carboxylic groups were introduced into the polymers. The scaffold surfaces were then modified via RGD peptide grafting. The powders of the synthesized mPEG-PCL diblock copolymers and HAp were mixed at a weight ratio of 60:40. FDM rapid prototyping technology was used to fabricate the scaffold by a home-made air pressure-aided deposition system. The home-made deposition system is composed of a stainless-steel sample container, a heating system, an air compressor, motors for *X*, *Y* and *Z* axes, and self-developed software (NI LabView 8.5, National Instruments Corporation, Austin, Texas, U.S). The mixed materials were melted by heating up to 60 °C. Subsequently, a pressure of 15 psi was applied to extrude the molten sample from a 0.4 mm nozzle onto a data processor-controlled *X*–*Y*–*Z* table. The construction of the scaffolds was computer-designed, and the extruded filament was laid down layer by layer in orientations of 0°/90°. The printing speed was hand-optimized to a feed rate of 4 mm/s. The average pore size measurements of the scaffolds were 100~150 μm as our previous study. The RP fabricated scaffolds were immersed in a solution of dimethylaminopropyl-3-ethylcarbodiimide hydrochloride (EDC, 0.2 M) + *N*-hydroxysuccinimide (NHS, 0.1 M) in (2-(*N*-morpholino)-ethanesulfonic acid (MES buffer, 0.1 M in dd H_2_O) for 1 h to activate the hydroxyl groups. Pure water was used to flush the scaffolds for 30 min. RGD peptides grafting was achieved via a condensation reaction by immersing the mPEG-PCL-COOH scaffolds in a solution of RGD peptides (10^–3^ M) and PBS for 24 h at 4 °C. Afterward, the scaffolds were rinsed with dd H_2_O (100 mL) for 10 min to remove non-grafted peptides and freeze-dried to remove water.

The physical and chemical properties and biological toxicity have been analyzed and the results was put in the Supplementary file. The analysis including Differential scanning calorimetry (DSC), Fourier transform infrared spectroscopy (FTIR) (Supplementary Figure S1), Gel Permeation Chromatography( GPC), nuclear magnetic resonance spectrophotometry (^1^H NMR) (Supplementary Figures S2 and S3), Thermogravimetric Analysis (TGA), In Vitro degradation (Supplementary Figure S4), and agar diffusion test for cytotoxicity analysis (Supplementary Figure S5).

### 4.2. Scaffold Implantation

The animal experimental protocol was approved by the Institutional Animal Experiment Committee of Chung Yuan Christian University and the Pigmodel^®^ Animal Technology Co., Ltd. (Miaoli, Taiwan) (project identification code: PIG-105007, 15 April 2016). All animal surgeries were performed in a certified operating room at the Pigmodel^®^ Animal Technology Co., Ltd., under general anesthesia using sterile techniques. Two male 2-year-old Lanyu miniature pigs weighing 25–28 kg were used for the evaluation of cartilage and bone regeneration. The pigs were put under general anesthesia, and their knees were shaved and disinfected with 2% chlorhexidine gluconate in 70% isopropyl alcohol. Operations performed were identical. Arthrotomy was made through a longitudinal medial parapatellar incision, and the femur condyle was exposed. A cylindrical osteochondral defect was created in the center of each medial femur condyle using an osteochondral coring device (OATS, Arthrex, Naples, FL, USA). A core 10 mm in diameter and 10 mm in depth was removed. Then, the scaffold was implanted into the defect and infiltrated with light-curing gel containing TGF-β1, cross-linking with 365 nm ultraviolet light for 10 min. In the control group, the defects were created without scaffold implantation.

### 4.3. Magnetic Resonance Imaging (MRI) and Computed Tomography (CT)

To monitor the progress of regeneration in a non-invasive method, magnetic resonance imaging (MRI) was performed 6 months after scaffold implantation under general anesthesia without any contrast agent. The minipigs were sacrificed, and joints were harvested one year after implantation. Computed tomography (CT) scans of the harvested knee joints were performed, and osteochondral specimens were evaluated pathologically.

### 4.4. Macroscopic Examination

One year after surgery, the animals were sacrificed for evaluation of the regeneration condition of the femoral condyles. Macroscopically, cartilage surface condition of the repair sites was observed and recorded. The repaired cartilage was scored using the ICRS scoring system for cartilage repair [40] (Table 2). 

### 4.5. Histological Evaluation

Each condyle was sectioned along the sagittal and frontal plane to evaluate the regeneration condition of the cartilage and subchondral bone. The specimens were fixed in 4% paraformaldehyde, decalcified in 10% Ethylenediaminetetraacetic acid, dehydrated, embedded in paraffin, sectioned to a 5 μm thickness, and stained with hematoxylin and eosin (H & E; Sigma, St. Louis, MO, USA), and Alcian blue. The regenerated cartilage quality was evaluated according to the Visual Histological Assessment Scale published by the ICRS [41]. The ICRS scale is based on the parameters of surface, matrix, cellular distribution, cell population viability, subchondral bone, and cartilage mineralization. High scores of the parameters indicate good-quality cartilage regeneration (Table 3).

## 5. Conclusions

In our study, we obtained demonstrated in vivo healing of an osteochondral defect implanted with biphasic scaffolds of poly(ε-caprolactone)/hydroxyapatite and glycidyl methacrylate-modified hyaluronic acid in a minipig. One year after the scaffold was implanted into the Lanyu miniature pig’s knee, the cartilage was regenerated successfully on the articular side of the scaffold and the morphology was significantly closer to hyaline cartilage compared to the control group. Although the scaffold was still not fully absorbed, bone tissue ingrowth to the pores of the scaffold was observed. Our study thus proposes a new clinical option to be considered alongside current treatments of cartilage injury.

## Figures and Tables

**Table 1:** The result of ICRS Visual Histological Assessment Scale.

Feature	Score of Experimental Group	Score of Control Group
Surface	2.5	0.5
Matrix	2	0.5
Cell distribution	2	0.5
Cell viability	2	2
Subchondral bone	2.5	1
Cartilage mineralization (calcification)	3	3

**Table 2:** International Cartilage Repair Society (ICRS) macroscopic evaluation of cartilage repair.

ICRS-Cartilage Repair Assessment System	Points
Degree of defect repair (Mossaicplasty; OAT; osteochondral allografts; others)	
100% survival of initially grafted surface	4
75% survival of initially grafted surface	3
50% survival of initially grafted surface	2
25% survival of initially grafted surface	1
0% (plugs are lost or broken)	0
Integration to border zone	
Complete integration with surrounding cartilage	4
Demarcating border < 1 mm	3
3/4th of graft integrated, 1/4th with a notable border >1 mm width	2
1/2 of graft integrated with surrounding cartilage, 1/2 with a notable border >1 mm	1
From no contact to 1/4th of graft integrated with surrounding cartilage	0
Macroscopic appearance	
Intact smooth surface	4
Fibrillated surface	3
Small, scattered fissures or cracs	2
Several, small or few but large fissures	1
Total degeneration of grafted area	0
Overall repair assessment	
Grade I: normal	12
Grade II: nearly normal	11–8
Grade III: abnormal	7–4
Grade IV: severely abnormal	3–1

**Table 3:** ICRS Visual Histological Assessment Scale, modified from the scale described by Mainil-Varlet et al. [41].

Feature	Score
Surface	
Smooth/continuous	3
Discontinuous/irregular	0
Matrix	
Hyaline cartilage	3
Hyaline cartilage/fibrocartilage	2
Fibrocartilage	1
Fibrous tissue	0
Cell distribution	
Columnar	3
Columnar/clustery	2
Clustery	1
Individual cells/disorganized	0
Cell viability	
Predominantly viable	3
Partially viable	1
Less than 10% viable	0
Subchondral bone	
Normal	3
Increased remodeling	2
Bone necrosis/granulation tissue	1
Detached/fracture/callus at base	0
Cartilage mineralization (calcification)	
Normal	3
Abnormal/inappropriate location	0

## Supplementary Materials

Supplementary materials can be found at http://www.mdpi.com/1422-0067/19/4/1125/s1.
