# Supplementary material for: Healing of Osteochondral Defects Implanted with Biomimetic Scaffolds of Poly(ε-Caprolactone)/Hydroxyapatite and Glycidyl-Methacrylate-Modified Hyaluronic Acid in a Minipig
Source: Int J Mol Sci. 2018 Apr 9;19(4):1125. doi: 10.3390/ijms19041125 (PMC5979374; doi:10.3390/ijms19041125)
Supplement: Supplementary file 1 [file ijms-19-01125-s001.pdf]

Supplementary Materials: Characterization of Biomimetic Scaffolds of Poly(ε-Caprolactone)/ Hydroxyapatite and Glycidyl Methacrylate-Modified Hyaluronic Acid

Yi-Ho Hsieh, Bo-Yuan Shen, Yao-Horng Wang, Bojain Lin, Hung-Maan Lee and Ming-Fa Hsieh

1. DSC Analysis

Differential scanning calorimetry (Jade DSC, PerkinElmer, Waltham, MA, USA) was used to study the thermal behavior of the synthesized mPEG-PCL and mPEG-PCL-COOH. Five to 10 mg samples were put into the aluminum pans. The samples were heated from 20 to 150 °C and then cooled down to 20 °C with rate of 10 °C/min.

The thermal behavior of the synthesized mPEG-PCL and mPEG-PCL-COOH was analysis by DSC. The results showed that the melting temperature of mPEG-PCL was 60.63 °C and the freezing temperature was 32.92 °C. The melting temperature of mPEG-PCL-COOH was 59.06 °C and the freezing temperature is 36.34 °C. There is no significant differences of DCS analysis between the mPEG-PCL and mPEG-PCL-COOH. According to the literatures, the melting temperature of PCL falls between 52 °C and 62 °C. The result indicated that the chemical modification of the terminal functional groups does not affect the characteristics of the fabric.

2. FTIR Spectra

Chemical bonds were identified by Fourier transform infrared spectroscopy (FTIR, Jasco, Tokyo, Japan) in the wave number ranging from 4000 to 450 cm^{-1}. The powdery polymers were mixed with potassium bromide (KBr) powder in a ratio of 1:100 for FTIR measurements.

The mPEG-PCL and mPEG-PCL-COOH biopolymers were analyzed by FTIR (Figure S1). The FTIR spectrum of mPEG-PCL and mPEG-PCL-COOH all have a strong absorption peak at 1731.76–1727.91 indicated the carbonyl group and peak at 1180.22 cm^{-1} associated with the C–O–C stretching. Furthermore, another absorption peak at 3444.24 cm^{-1} presented at the mPEG-PCL-COOH FTIR spectrum indicated the terminal function group of hydroxyl (OH–) stretching in the carboxyl group. According to the literature, the characteristic absorption peaks of C=O and CH of the PCL chain are 1725 cm^{-1} and 2936 cm^{-1}, respectively, and the peak of the C–O–C absorption on the mPEG chain is 1100 cm^{-1}. The results indicated that the synthesized materials were mPEG-PCL and mPEG-PCL-COOH.

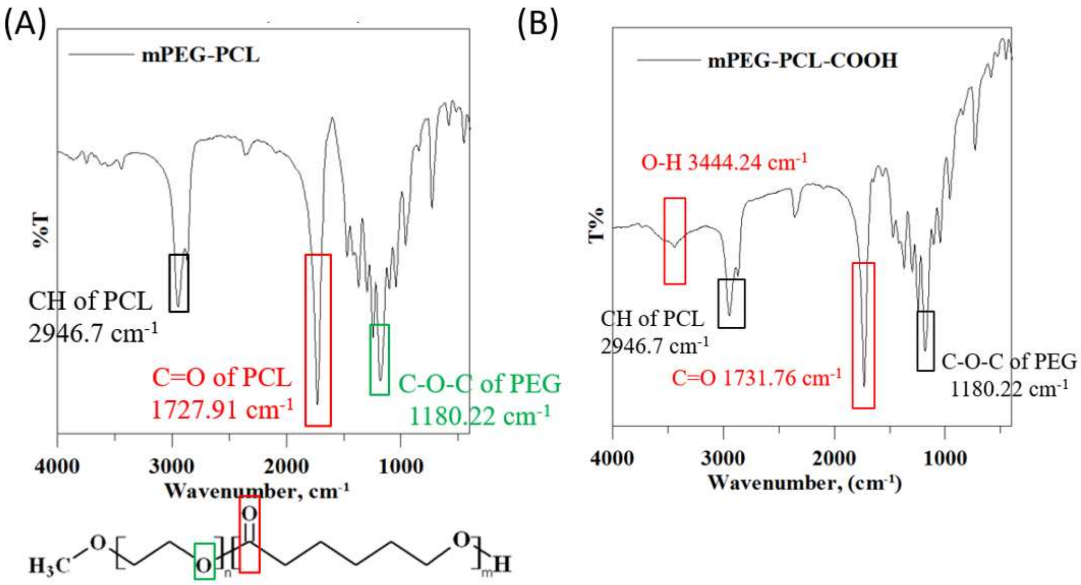

Figure S1. The FTIR spectra of (A) mPEG-PCL and (B) mPEG-PCL-COOH.

3. GPC Analysis

Gel Permeation Chromatography (GPC 270, Viscotek, Malvern, UK) analysis was performed for the synthesized polymers to determine the number average molecular weight (Mn), the weight average molecular weight (Mw) and polydispersity index (PDI). Using tetrahydrofuran (THF) as the solvent.

Molecular weight and molecular weight distribution were characterized by GPC and ^{1}H NMR. As mentioned in our previous study, the molecular weight and polydispersity (PDI, Mw/Mn) of mPEG-PCL-COOH polymer is 9514.33 ± 389.70 (Da) and 1.19 ± 0.01.

4. ^{1}H NMR Spectra

The organic chemical components of the polymer were determined by nuclear magnetic resonance spectrophotometry (500 MHz, Bruker, MA, USA). All samples were measured and recorded as solutions in deuterated chloroform.

The ^{1}H NMR spectra of mPEG-PCL and mPEG-PCL-COOH were shown in Figure S2. CDCl3 was used as the solvent and the signal of 7.26 ppm was detected. As seen in the spectrum of mPEG-PCL and mPEG-PCL-COOH, the resonance signals at 1.36, 1.65, 2.31, and 4.06 ppm are attributed to the chemical shift of the methylene protons of –(CH2) 3–, –OCCH2– and –CH2OOC– in the PCL unit. The PEG unit shows its characteristic resonance signals at 3.37 and 3.64 ppm, which are associated with CH3– and–CH2CHO–. The spectra of mPEG-PCL-COOH showed the carboxyl group at 2.65 ppm.

(A)

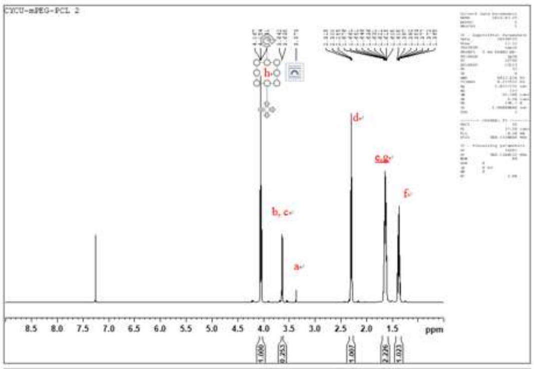

^{1}H-NMR spectrum of mPEG-PCL 9450

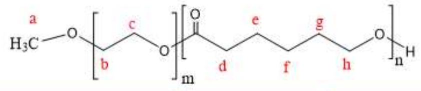

Group	Peak (ppm)	Area
-CH_3O (a)	3.37	
-CH_2CH_2O (b, c)	3.64	0.253
-CH_2 (d)	2.31	1.007
-CH_2 (e,g)	1.65	2.226
-CH_2 (f)	1.36	1.023
-CH_2 (h)	4.06	1

(B)

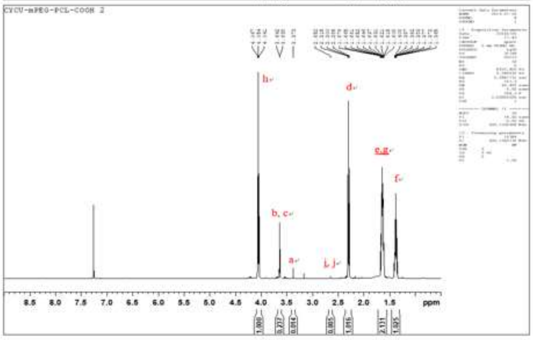

^{1}H-NMR spectrum of mPEG-PCL-COOH9450

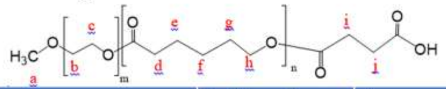

Group	Peak (ppm)	Area
-CH_3O (a)	3.37	0.014
-CH_2CH_2O (b, c)	3.64	0.237
-CH_2 (d)	2.31	1.016
-CH_2 (e,g)	1.65	2.131
-CH_2 (f)	1.36	1.025
-CH_2 (h)	4.06	1
-CH_2 (i,j)	2.65	0.005

Figure S2. 1H NMR spectrum of (**A**) mPEG-PCL and (**B**) mPEG-PCL-COOH.

The ^{1}H NMR spectra of GMHA (Figure S3). The two peaks at 5.8 and 6.5 ppm are associated with the methacrylate groups (c,b), while the methylgroup of HA appears at 2 ppm (a).

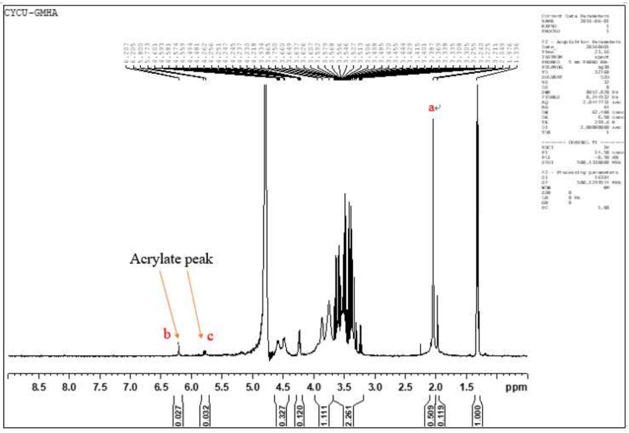

GMHA

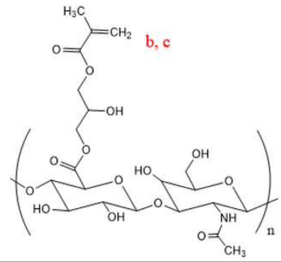

Groups	Peak (ppm)	Area
HA	3 – 4	1.111 + 2.261
Acrylates in GM (b, c)	6.2 and 5.8	0.027 + 0.032
HA methyl group (a)	2	0.509 + 0.119

Figure S3. ^{1}H NMR spectrum of GMHA.

5. TGA Analysis

Thermogravimetric Analysis (TGA) is used to determine the inorganic composition of the materials and to predict their thermal stability. Samples with weights ranging from 10 to 20 mg were put into the furnace and heated from 25 °C to 900 °C. All operations were carried on at the rate of 10 °C/min.

From thermogravimetric analysis (TGA), it was observed that the onset of thermal degradation started at about 300 °C, and at 400 °C entire amount of PCL had degraded. In pure HAp, no thermal degradation was observed due to the thermal degradation temperature is in the range between 1360 °C to 1400 °C. As witnessed in the TGA of the scaffolds, the weight percentage that was left in the pan indicated the composition of HAp, which was 35 wt % and comparable to our design.

6. In Vitro Degradation

To ensure an appropriate bone restoration, the selected material must have a degradation rate that's close to the new bone formation. The in vitro degradation behavior in phosphate buffer solution (PBS) according to the standard protocol ASTM F1635 was investigated. The sterilized RP scaffolds were placed in 100 mL glass container containing 50 mL of PBS and incubated at 37 °C shaking with 50 rpm for three months. The characterization and morphological analysis of the degraded scaffolds were performed

After 12 weeks of immersion in PBS, scaffolds exhibited the rough surface with HAp exposure. The image of the scaffolds clearly illustrates the complete cross section of the scaffold matrix is affected by erosion, the white dots on the fracture and erosion surface was noted, too (Figure S4). According to the phenomenon appeared on the fracture and erosion surfaces, the scaffold underwent both bulk and surface erosion was suspected. After 3 months of degradation, the weight of the scaffold decreased to 20% of its original value.

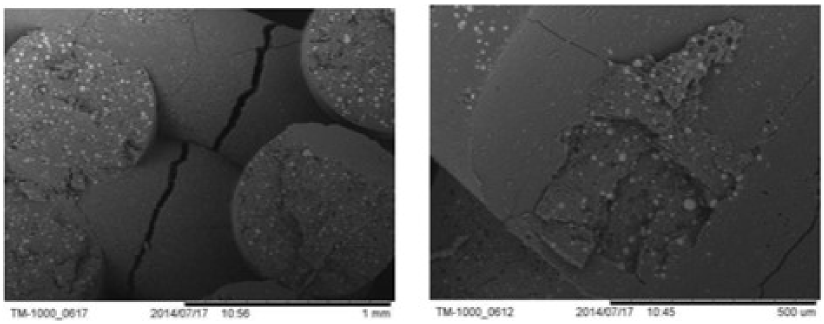

Figure S4. Scanning Electron Microscope Imaging of the scaffold after immersed in PBS solution for 12 weeks.

7. Cytotoxicity of the Scaffold and Hydrogel

The agar diffusion test on L929 mouse fibroblast cells was used for cytotoxicity analysis. The scaffold (PEG-PCL-RGD) and the hydrogel (GMHA) test materials were placed on the agar, Latex Gloves were used for the material of the positive control group and Teflon was used as a negative control group. Cytotoxicity was observed by measuring the zones of decolorization and evaluating cell lysis under microscope using the established criteria after 24 h.

The cytotoxicity result of agar diffusion test of the scaffold (PEG-PCL-RGD) and the hydrogel (MGHA) are presented in Figure S5. Both the scaffold and the hydrogel showed the decolorization score of 2 and the cell lysis score of 2.

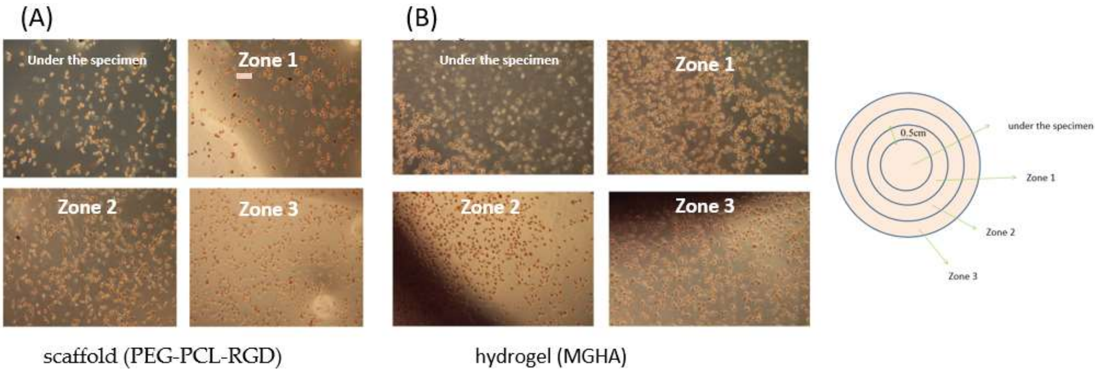

Figure S5. 24 h L929 agar diffusion for PEG-PCL-RGD (**A**) and MGHA (**B**).

A Teflon mold was used as a negative control with no cellular lysis. The latex glove was used as a positive control and resulted in a decolorization score of 4 and cell lysis score of 5. The result indicated that the scaffold (PEG-PCL-RGD) and the hydrogel (MGHA) were low toxicity and cells could grow and proliferation in the environment with the synthetic materials.
